# A dynamic prediction model for prognosis of acute-on-chronic liver failure based on the trend of clinical indicators

**DOI:** 10.1038/s41598-021-81431-0

**Published:** 2021-01-19

**Authors:** Zhenjun Yu, Yu Zhang, Yingying Cao, Manman Xu, Shaoli You, Yu Chen, Bing Zhu, Ming Kong, Fangjiao Song, Shaojie Xin, Zhongping Duan, Tao Han

**Affiliations:** 1 Department of Hepatology and Gastroenterology, The Third Central Clinical College of Tianjin Medical University, No. 83, Jintang Road, Hedong District, Tianjin, 300170 China; 2 Liver Disease Center (Difficult & Complicated Liver Diseases and Artificial Liver Center), Beijing You’an Hospital Affiliated to Capital Medical University, Beijing, China; 3 Liver Failure Treatment and Research Center, The Fifth Medical Center of Chinese, PLA General Hospital, Beijing, China; 4 Department of Hepatology and Gastroenterology, Tianjin Third Central Hospital Affiliated to Nankai University, Tianjin, China; 5 Tianjin Key Laboratory of Extracorporeal Life Support for Critical Diseases, Artificial Cell Engineering Technology Research Center, Tianjin Institute of Hepatobiliary Disease, The Tianjin Third Central Hospital, Tianjin, China

**Keywords:** Hepatology, Prognostic markers

## Abstract

Acute-on-chronic liver failure (ACLF) is a dynamic syndrome, and sequential assessments can reflect its prognosis more accurately. Our aim was to build and validate a new scoring system to predict short-term prognosis using baseline and dynamic data in ACLF. We conducted a retrospective cohort analysis of patients with ACLF from three different hospitals in China. To construct the model, we analyzed a training set of 541 patients from two hospitals. The model’s performance was evaluated in a validation set of 130 patients from another center. In the training set, multivariate Cox regression analysis revealed that age, WGO type, basic etiology, total bilirubin, creatinine, prothrombin activity, and hepatic encephalopathy stage were all independent prognostic factors in ACLF. We designed a dynamic trend score table based on the changing trends of these indicators. Furthermore, a logistic prediction model (DP-ACLF) was constructed by combining the sum of dynamic trend scores and baseline prognostic parameters. All prognostic scores were calculated based on the clinical data of patients at the third day, first week, and second week after admission, respectively, and were correlated with the 90-day prognosis by ROC analysis. Comparative analysis showed that the AUC value for DP-ACLF was higher than for other prognostic scores, including Child–Turcotte–Pugh, MELD, MELD-Na, CLIF-SOFA, CLIF-C ACLF, and COSSH-ACLF. The new scoring model, which combined baseline characteristics and dynamic changes in clinical indicators to predict the course of ACLF, showed a better prognostic ability than current scoring systems. Prospective studies are needed to validate these results.

## Introduction

Acute-on-chronic liver failure (ACLF) is a clinical syndrome characterized by acute decompensation of chronic liver disease, and often triggered by acute strikes or precipitating events^1^. It can be accompanied by multiple organ failures, and poses a major threat to public health^2^. Additionally, ACLF is an extraordinarily dynamic syndrome that can either improve or even completely resolve in up to 50% of cases, while in others it may deteriorate to a life-threatening condition due to disease progression^3^. Due to the high mortality rate, accurate prognosis prediction in ACLF has always been a hot topic in liver research. Early recognition of the poor prognosis in ACLF is not only helpful to minimize ineffective and expensive treatments, but also for rational allocation of liver transplantation resources^4^.

Although a variety of scoring systems have been developed to assess prognosis in ACLF, most of them are based on clinical indicators at the time of diagnosis. Examples are the classic Child–Turcotte–Pugh (CTP) and Model of End-stage Liver Disease (MELD) score^5,6^, or the more recent Chronic Liver Failure Sequential Organ Failure Assessment (CLIF-SOFA) and Chronic Liver Failure Consortium Acute-on-Chronic Liver Failure (CLIF-C ACLF) score proposed by the European Association for the Study of Chronic Liver Failure^7,8^. In recent years, research has focused on dynamic assessments that reflect more accurately the clinical condition and prognosis of ACLF. For example, Gustot et al.^9^ evaluated the clinical course by comparing the CLIF-C ACLF scores of patients at different time points, and found a close correlation between prognosis and changes in clinical condition. Most of the patients would have a clear prognosis between day 3 and 7 of hospital admission and clinical decisions such as evaluation for liver transplant or discussion over goals of care could be tailored using clinical scores. The ACLF guidelines proposed by the Asian Pacific Association for the Study of the Liver (APASL)^10^ recommend that ACLF patients should be assessed with the APASL ACLF Research Consortium (AARC)-ACLF score on admission and dynamically evaluated on the 4th and 7th day of treatment to predict progression. The cumulative mortality increases with rises in the AARC-ACLF score in the first week^10^.

Due to its rapidly changing nature, both initial characteristics and dynamic trends of clinical indicators are helpful to predict prognosis in ACLF^11^. Sequential assessment at multiple time-points may accurately reflect the clinical course and the responsiveness to medical treatment, and theoretically improve the prognostic ability compared to a single time-point^12^. This research aimed to build a dynamic prediction model of ACLF using baseline and dynamic clinical indicators, with the purpose to provide a basis for the development of individualized treatment.

## Materials and methods

### Study design, participants, and data collection

This was a multicenter retrospective cohort study performed in China (ChiCTR1900021539). Two groups of patients were analyzed: one for building a predictive model (training set) and the other for model validation (validation set). The training group included patients admitted to the Tianjin Third Central Hospital and the Fifth Medical Center of PLA General Hospital between November 1, 2012 and June 30, 2019. The validation group included patients admitted to Beijing You’an Hospital Affiliated to Capital Medical University between January 1, 2015 and June 30, 2019.

Owing to the overlap of terminologies, more than a dozen definitions have emerged to describe ACLF, and the most widely accepted ones are from the APASL and a joint conference of the European Association for the Study of the Liver and the American Association for the Study of Liver Diseases^13^. Considering the differences between Western and Eastern definitions, the World Gastroenterology Organization (WGO) established a new definition, which divided ACLF into three categories based on the status of chronic liver disease: type-A for patients without cirrhosis, type-B for well compensated cirrhosis and type-C for previous hepatic decompensation^14^. According to the WGO classification, the inclusion criteria for ACLF cases in this study were as follows: on the basis of chronic liver disease, a liver failure occurring and manifested as jaundice (serum total bilirubin [TB] ≥ 5 mg/dl) and coagulation dysfunction (international normalized ratio [INR] ≥ 1.5 or prothrombin activity [PTA] < 40%) within 4 weeks.

Exclusion criteria were as follows: (1) human immunodeficiency virus infection, (2) severe extrahepatic chronic disease, mainly refers to primary heart failure, severe chronic pulmonary disease, and chronic kidney disease requiring renal replacement therapy, etc. (3) Liver cancer or other malignancy, and (4) pregnancy.

Artificial liver support system (ALSS) treatment was performed in either single or combination mode. The single-mode involved plasma exchange, and the combination mode consisted of plasma exchange together with the double plasma molecular adsorption system treatment or with hemofiltration in patients with hepatic encephalopathy or acute kidney injury. Depending on the patient’s condition, different treatment modalities for ALSS were chosen, with treatment times ranging from one to three times per week.

All data on patients were retrieved from manual and electronic medical records. All study procedures followed the principles of the Declaration of Helsinki. Being a retrospective study, this work was approved and the need for informed consent was waived by the Ethics Committee of the Tianjin Third Central Hospital, Beijing You'an Hospital Affiliated to Capital Medical University, and the Fifth Medical Center of PLA General Hospital.

### Scoring models

Comparison with the following scoring models was performed: CTP^5^, MELD^6,15^, MELD-sodium (MELD-Na)^16^, CLIF-SOFA^7^, CLIF-C ACLF^8^, and a prognostic scoring model for hepatitis B virus-related ACLF (HBV-ACLF) proposed by the Chinese Group on the Study of Severe Hepatitis B (COSSH-ACLF)^17^. For a detailed description of these scoring systems, please refer to Supplementary Information.

### Statistical analysis

Continuous data were expressed as median (interquartile range), and properly analyzed by t-test, one-way Anova, and non-parametric Mann–Whitney U test. Categorical variables were expressed in frequency (percentage) and compared by chi-square or Fisher precision tests. Multivariate Cox regression analysis was used to determine the independent prognostic factors. In multivariate analysis, the binary logistic regression equation was constructed according to the forward likelihood ratio test. The areas under the ROC curve (AUC) of various prognostic scoring systems were compared using Delong’s z-test. Univariate Cox regression of Harrell's C /Somer's D indexes was used to judge the fitting degree of survival model, and the Kaplan–Meier method was used to compare the cumulative survival rate. All tests were double-tailed, and a *p* value < 0.05 was considered statistically significant. Statistical analysis and mapping were performed using IBM SPSS Statistics (version 22.0) (IBMCorp, North Castle, New York, USA), R (version 3.6.3)^18^, with rms and survival packages (Foundation for Statistical Computing, Vienna, Austria) (https://www.R-project.org), and Python (version 3.7.6), with matplotlib and sklearn packages (Python Software Foundation, Beaverton, USA) (https://www.python.org).

## Results

### Baseline characteristics of training set cases

**Figure 1:**
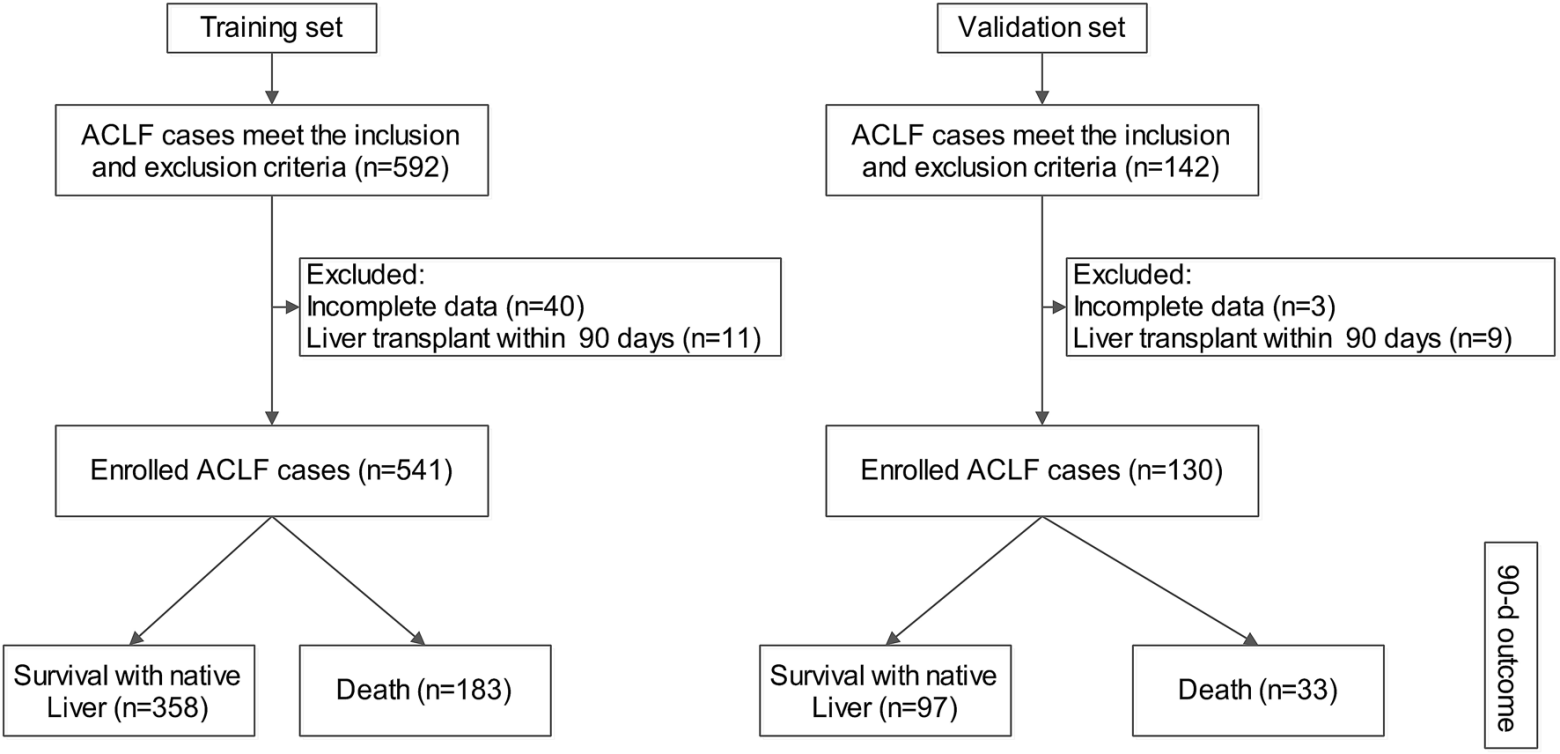
Flowchart of ACLF training and validation group cases screening.

A total of 592 cases meeting the inclusion and exclusion criteria were collected, 40 cases with incomplete data and 11 cases with liver transplantation within 90 days were further excluded. The remaining 541 patients (356 patients from the Tianjin Third Central Hospital and 185 patients from the Fifth Medical Center of PLA General Hospital), were enrolled in the training group. Among them, there were a total of 453 patients with cirrhosis (83.7%), and 183 cases (33.8%) died within 90 days (Fig. 1). Supplementary Table S1 describes the baseline characteristics of the training group upon admission. Most patients were males and had a history of cirrhosis, with HBV infection as the most frequent etiology, followed by alcoholic liver disease. More than half of patients had unclear or no obvious precipitating events.

**Figure 2:**
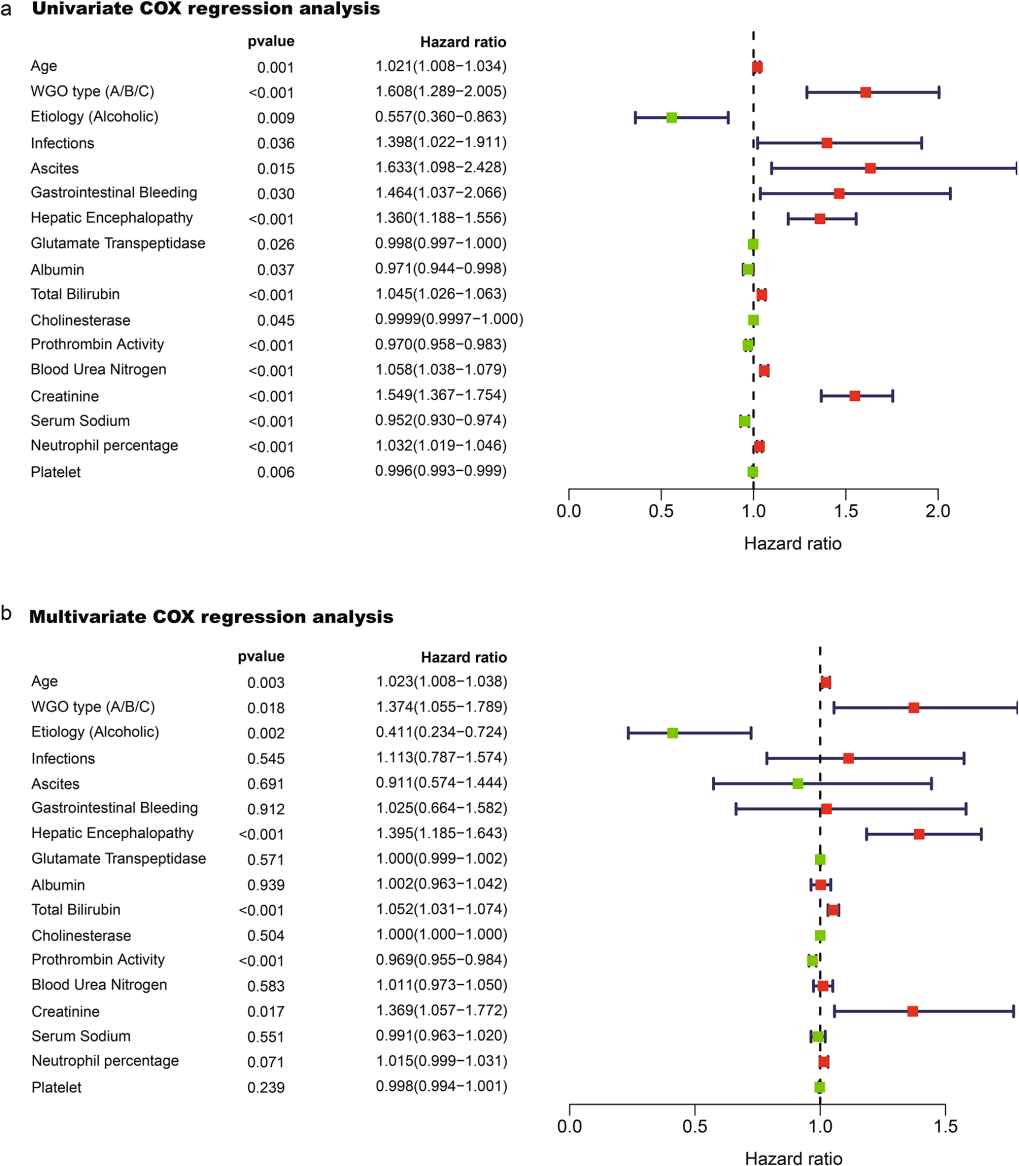
(**a**) Univariate Cox regression analysis showing that the factors related to ACLF prognosis were age, WGO type, basic etiology, bacterial infection, ascites, gastrointestinal bleeding, hepatic encephalopathy stage, glutamate transpeptidase, albumin, total bilirubin, cholinesterase, prothrombin activity, blood urea nitrogen, creatinine, serum sodium, neutrophil ratio, and platelets. (**b**), Multivariate Cox regression analysis confirmed that age, WGO type, alcoholic etiology, total bilirubin, creatinine, prothrombin activity, and hepatic encephalopathy stage remained associated with prognosis of ACLF. (Assignment of indexes in COX regression analysis was as follows: WGO type A, B, and C of ACLF were assigned 1, 2, and 3 respectively; basic etiology was assigned 1 for the alcoholic liver disease alone, and 0 for others; positive complications including bacterial infection, gastrointestinal bleeding, ascites were all assigned 1, no complications 0; hepatic encephalopathy was assigned 0, 1, 2, 3, and 4 according to the West Haven stages 0, I, II, III, and IV, respectively; artificial liver system support (ALSS) was assigned 1, no ALSS 0).

Enrolled ACLF cases were classified into three different types: type A (without cirrhosis), type B (compensated cirrhosis), and type C (decompensated cirrhosis). Prognosis at 90 days differed significantly depending on the WGO type (χ^2^ = 20.800, *p* < 0.001), with the mortality rates of 44.4% in type C ACLF cases and 25.0%, 26.0% in type A and B ACLF cases, respectively. Notably, we found that prognosis of type C patients with alcoholic liver disease alone was significantly better than that of patients with HBV infection or HBV infection combined with alcoholic liver disease (χ^2^ = 9.799, *p* = 0.007); while similar differences were absent in patients with type A or B ACLF (Table 1). Factors related to ACLF prognosis in Univariate Cox analysis were shown in Fig. 2a. Multivariate Cox regression analysis showed that age, WGO type, alcoholic etiology, TB, serum creatinine (Cr), PTA, and hepatic encephalopathy (HE) (defined by the West-Haven criteria^19^) were all independent prognostic factors of ACLF (Fig. 2b).

**Table 1:** Prognostic impact of HBV infection and alcoholic liver disease as basic etiologies in patients with ACLF, stratified by WGO types A, B, and C.

WGO type	Etiology	Survivor	Non-survivor	χ^2^ value	*p* value
A	Alcoholic, n(%)	6 (75.0%)	2 (25.0%)	1.475	0.559
	HBV, n(%)	45 (75.0%)	15 (25.0%)		
	HBV and Alcoholic, n(%)	2 (50.0%)	2 (50.0%)		
B	Alcoholic, n(%)	40 (78.4%)	11 (21.6%)	1.199	0.576
	HBV, n(%)	80 (72.1%)	31 (27.9%)		
	HBV and Alcoholic, n(%)	10 (66.7%)	5 (33.3%)		
C	Alcoholic, n(%)	33 (76.7%)	10 (23.3%)	9.799	0.007
	HBV, n(%)	66 (50.0%)	66 (50.0%)		
	HBV and Alcoholic, n(%)	13 (50.0%)	13 (50.0%)		

*HBV* Hepatitis B virus.

In the training set, 37.9% of the patients were treated with an ALSS treatment. Regarding the effect of ALSS on prognosis, we performed the Univariate Cox regression analysis, which uncovered no significant difference in short-term prognosis of ACLF patients (*p* > 0.05). Further stratified analysis was performed according to the age, sex, etiology, WGO type, MELD score and complications, the statistical results showed that there was no significant difference in the mortality of cases in each group with or without ALSS treatment (*p* > 0.05, respectively) (Supplementary Table S2).

### Development of the dynamic prediction model for prognosis of ACLF (DP-ACLF)

**Figure 3:**
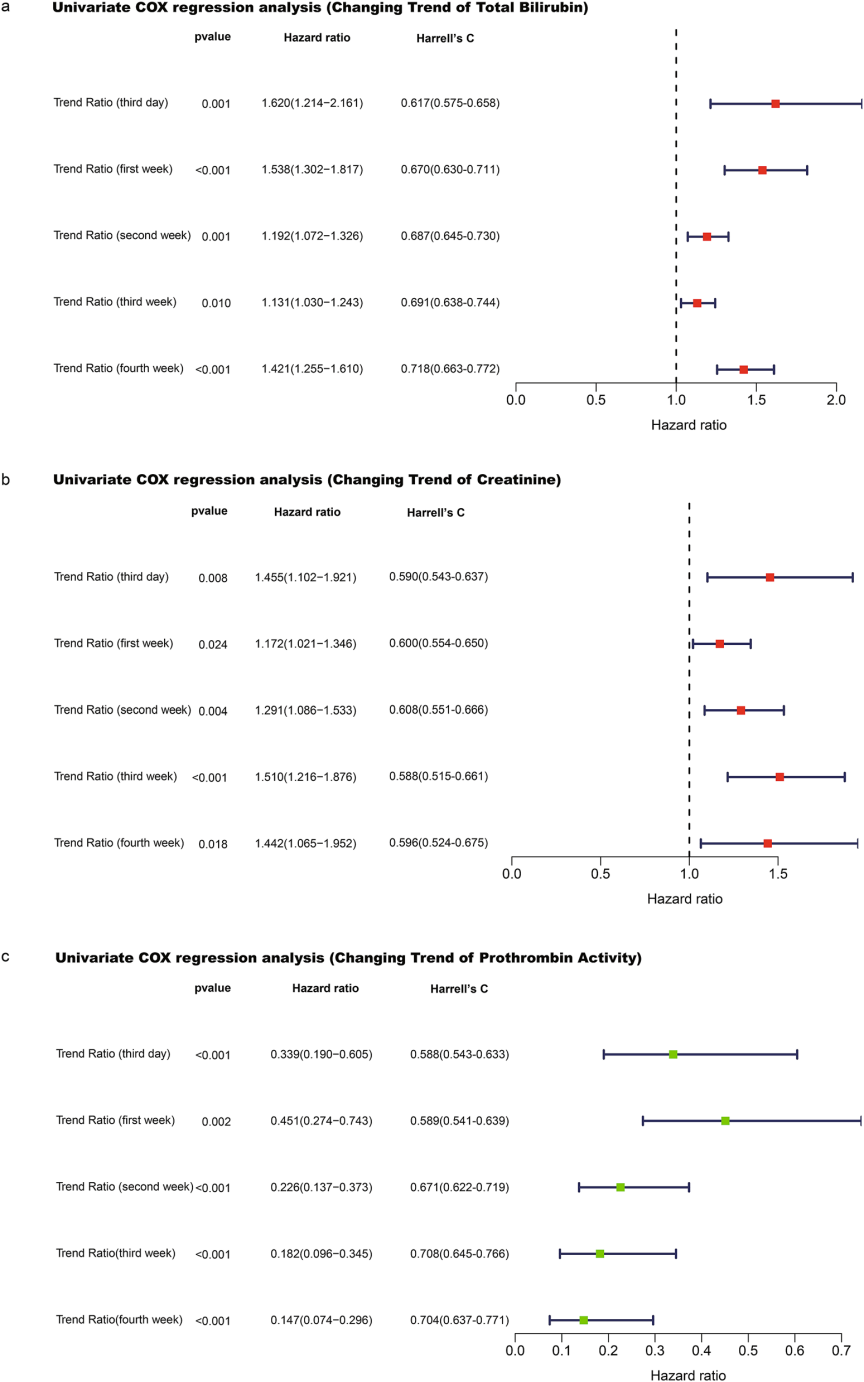
(**a**) Elevated levels of total bilirubin at the third day and first, second, third, and fourth weeks after admission were risk factors for death. The Harrell’s C index of model fitting degree showed an increasing trend with the extension of time. (**b**) Elevated levels of creatinine at the third day and first, second, third, and fourth weeks after admission were risk factors for death. The Harrell’s C index of model fitting degree increased to the highest value at the second week, and decreased slightly at other time points. (**c**) Declined levels of prothrombin activity at the third day and first, second, third, and fourth weeks after admission were all risk factors for death. The Harrell’s C index of model fitting degree showed an increasing trend with the extension of time.

To determine whether the evolution of clinical indicators was related to the prognosis of ACLF, we analyzed the dynamic changes in independent prognostic factors (TB, Cr, and PTA). Temporal variation of these parameters was assessed at the third day and first, second, third, and fourth weeks after admission; Univariate Cox regression analysis was performed. We found that increases in TB and Cr and decreases in PTA at all time points were risk factors of death (Fig. 3a–c). Considering that 3–4 weeks may be an excessively long period for clinical observation, we chose the third day, first week, and second week as the time nodes for calculating the changing trends of the parameters.

In order to facilitate trend analysis of prognostic variables, we developed a dynamic trend score table. An increase in TB or Cr, a decrease in PTA of more than 30%, and the aggravation of HE stage were assigned 3 points; a decrease in TB or Cr, an increase in PTA of more than 30%, and the improvement of HE stage were assigned 1 point. The detailed scoring criterias were shown in Table 2. To obtain a dynamic trend score model with the best fitting degree of survival time, the variation ranges of TB, Cr, and PTA were set at 10–50%, and the variation range of HE stage was set at 1–3 levels previously. Univariate Cox regression was used to calculate the corresponding variation range when each model of parameter score reached the best: most Harrell’s C indexes were at their best when variation ranges were set at 30% for TB, Cr, and PTA, and when HE stage variated one level (Supplementary Tables S3–5). Multivariate Cox regression analysis showed that the dynamic trend scores of TB, Cr, PTA and HE were all independent prognostic factors of ACLF (Supplementary Fig. S1).

**Table 2:** Dynamic trend scores based on the changing trends of total bilirubin, creatinine, prothrombin activity, and hepatic encephalopathy stage.

Dynamic trend score	1	2	3
Total Bilirubin trend	Decreased level > 30%	Increased or decreased level ≤ 30%	Increased level > 30%
Creatinine trend	Decreased level > 30%	Increased or decreased level ≤ 30%	Increased level > 30%
Prothrombin activity trend	Increased level > 30%	Increased or decreased level ≤ 30%	Decreased level > 30%
Hepatic Encephalopathy trend (West Haven stage)	Improved level ≥ 1	Unchanged	Aggravated level ≥ 1

To develop a new 90-day prognosis prediction model, we calculated the sum of dynamic trend scores (SDTs) of each patient, performed binary logistic regression analysis in combination with other independent prognostic factors, and obtained the optimal prediction model parameters and OR values through a forward progressive elimination process: age (1.034), WGO type (1.385), alcoholic etiology (0.511), TB (1.085), Cr (2.492), PTA (0.960), and SDTs (2.263) (Table 3). The obtained formula for DP-ACLF was as follows: DP-ACLF = 0.033 × age (years) + 0.326 × WGO type + 0.082 × TB (mg/dL) + 0.913 × Cr (mg/dL) + 0.817 × SDTs − 0.04 × PTA (%) − 0.672 × Etiology.

**Table 3:** Logistic regression analysis of 90-day survival rates.

Variable	B	SE	Wald χ^2^	*p* value	OR (95% CI)
Age	0.033	0.010	10.353	0.001	1.034 (1.013–1.055)
WGO type (A/B/C)	0.326	0.162	4.040	0.044	1.385 (1.008–1.902)
Etiology (alcoholic)	− 0.672	0.314	4.586	0.032	0.511 (0.276–0.945)
Total bilirubin	0.082	0.015	31.545	< 0.001	1.085 (1.054–1.116)
Creatinine	0.913	0.211	18.727	< 0.001	2.492 (1.648–3.768)
Prothrombin activity	− 0.040	0.010	18.075	< 0.001	0.960 (0.943–0.978)
Sum of dynamic trend scores (SDTs)	0.817	0.093	77.088	< 0.001	2.263 (1.886–2.715)
Constant	− 10.441	1.128	85.702	< 0.001	

*SDTs* Sum of dynamic trend scores.

(Etiology was assigned 1 for alcoholic liver disease alone, and 0 for others).

**Figure 4:**
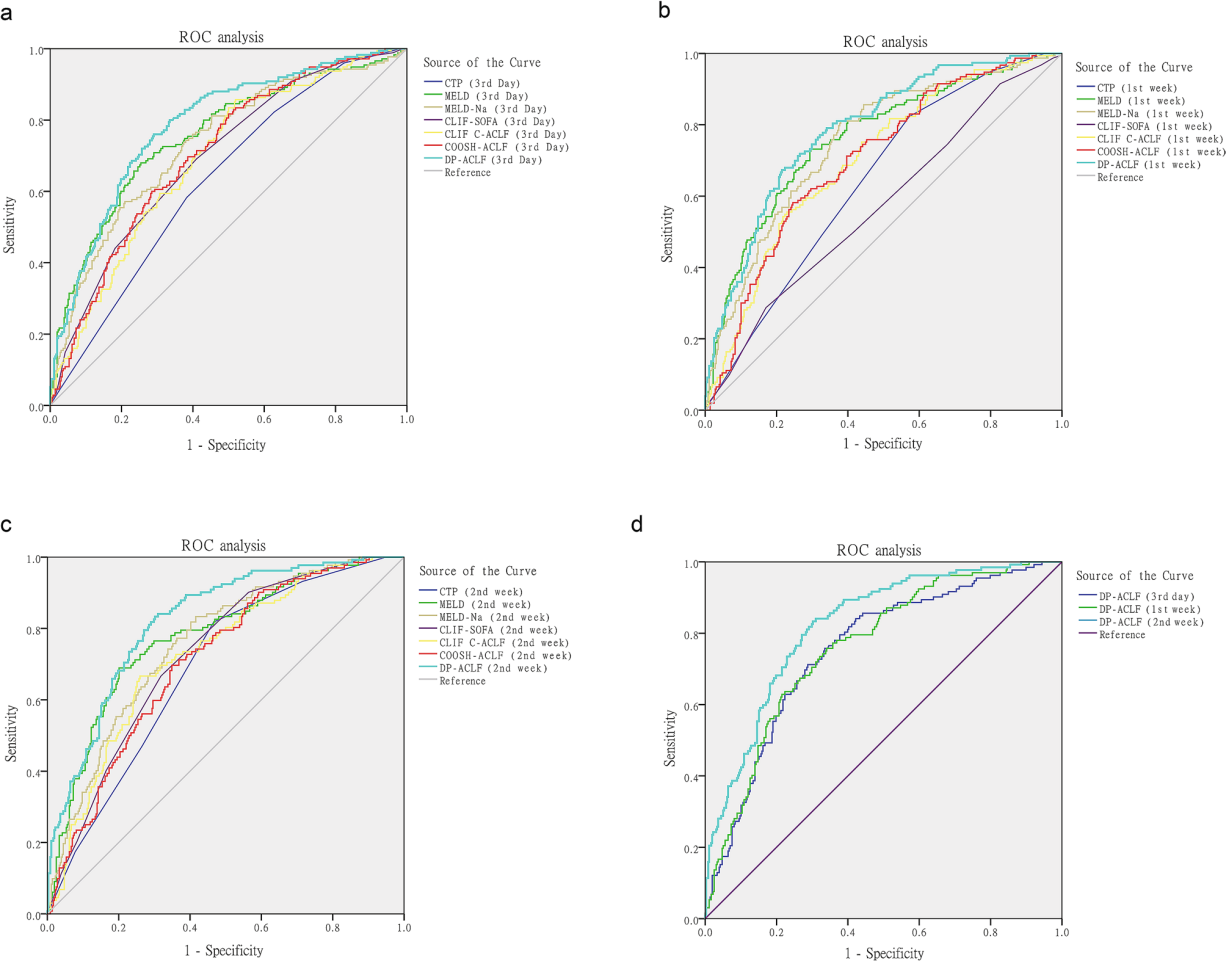
(**a–c**) DP-ACLF scores based on the patients of the training set at the third day, first week, and second week after admission, respectively, and comparison of ROC curves with other prognostic scores at the same time point. (**d**) ROC curve comparisons of DP-ACLF scores at different time points.

In patients with a survival time of more than 3 days (n = 533), each score was assessed at the third day after admission, and ROC curve comparisons showed AUC values as follows: DP-ACLF(0.787), CTP (0.632), MELD (0.756), MELD-Na (0.733), CLIF-SOFA (0.697), CLIF C ACLF (0.693), and COSSH-ACLF (0.706). The DP-ACLF had significantly higher AUCs than other scores at the third day (*p* < 0.05, respectively) (Table 4, Fig. 4a). And in patients with a survival time of more than 7 days (n = 511), each score was assessed at the first week after admission, and ROC curve comparisons showed AUC values as follows: DP-ACLF(0.788), CTP (0.642), MELD (0.760), MELD-Na (0.752), CLIF-SOFA (0.572), CLIF C ACLF (0.703), and COSSH-ACLF (0.703). The prognostic efficacy for DP-ACLF was significantly higher than for other scores at the first week (*p* < 0.05, respectively), except for the MELD and MELD-Na scores. (Table 4, Fig. 4b).

**Table 4:** Training group: ROC correlation analysis and comparison of DP-ACLF and other prognostic scores at the third day, first week and second week after admission.

Time point (cases, n)	Variable	AUC (95% CI)	St	Cut-off value	Sensitivity	Specificity	*z statistic (*p* value)
The third day after admission (n = 533)	CTP (3rd day)	0.632 (0.590–0.673)	0.024	12	0.583	0.617	5.595 (*p* < 0.001)
	MELD (3rd day)	0.756 (0.717–0.791)	0.023	25	0.680	0.746	2.171 (*p* = 0.030)
	MELD-Na (3rd day)	0.733 (0.694–0.770)	0.023	26	0.743	0.620	2.975 (*p* = 0.003)
	CLIF-SOFA (3rd day)	0.697 (0.656–0.736)	0.023	8	0.691	0.589	4.209 (*p* < 0.001)
	CLIF-C ACLF (3rd day)	0.693 (0.652–0.732)	0.024	39	0.857	0.480	3.944 (*p* < 0.001)
	COSSH-ACLF (3rd day)	0.706 (0.665–0.744)	0.023	6	0.817	0.500	3.549 (*p* < 0.001)
	DP-ACLF (3rd day)	0.787 (0.749–0.821)	0.021	9.7	0.760	0.710	NA
The first week after admission (n = 511)	CTP (1st week)	0.642 (0.598–0.683)	0.025	11	0.824	0.427	5.229 (*p* < 0.001)
	MELD (1st week)	0.760 (0.721–0.797)	0.024	23	0.732	0.704	1.702 (*p* = 0.089)
	MELD-Na (1st week)	0.752 (0.712–0.789)	0.023	25	0.811	0.620	1.796 (*p* = 0.073)
	CLIF-SOFA (1st week)	0.572 (0.528–0.615)	0.027	9	0.288	0.830	6.514 (*p* < 0.001)
	CLIF-C ACLF (1st week)	0.703 (0.661–0.742)	0.025	44	0.556	0.771	3.446 (*p* < 0.001)
	COSSH-ACLF (1st week)	0.703 (0.662–0.743)	0.024	6	0.582	0.754	3.614 (*p* < 0.001)
	DP-ACLF (1st week)	0.788 (0.750–0.823)	0.021	10	0.673	0.785	NA
The second week after admission (n = 490)	CTP (2nd week)	0.691 (0.648–0.732)	0.025	11	0.818	0.534	6.044 (*p* < 0.001)
	MELD (2nd week)	0.779 (0.739–0.815)	0.024	24	0.689	0.799	2.482 (*p* = 0.013)
	MELD-Na (2nd week)	0.759 (0.718–0.796)	0.023	23	0.833	0.584	3.017 (*p* = 0.003)
	CLIF-SOFA (2nd week)	0.728 (0.687–0.767)	0.024	8	0.667	0.682	5.230 (*p* < 0.001)
	CLIF-C ACLF (2nd week)	0.729 (0.687–0.768)	0.025	42	0.667	0.740	4.814 (*p* < 0.001)
	COSSH-ACLF (2nd week)	0.712 (0.669–0.752)	0.025	6	0.697	0.651	5.089 (*p* < 0.001)
	DP-ACLF (2nd week)	0.823 (0.786–0.856)	0.020	9.4	0.833	0.698	NA

In patients with a survival time of more than 14 days (n = 490), each score was assessed at the second week after admission, and ROC curve comparisons showed AUC values as follows: DP-ACLF(0.823), CTP (0.691), MELD (0.779), MELD-Na (0.759), CLIF-SOFA (0.728), CLIF C ACLF (0.729), and COSSH-ACLF (0.712). The DP-ACLF had significantly higher AUCs than other scores at the second week (*p* < 0.05, respectively) (Table 4, Fig. 4c). Furthermore, comparing the predictive efficacy of DP-ACLF scores at different time points in patients showed that the AUC values for DP-ACLF (2nd week) were significantly higher than those for DP-ACLF (3rd day) (z = 3.524, *p* < 0.001) and DP-ACLF (1st week) (z = 3.449, *p* < 0.001) (Fig. 4d).

**Figure 5:**
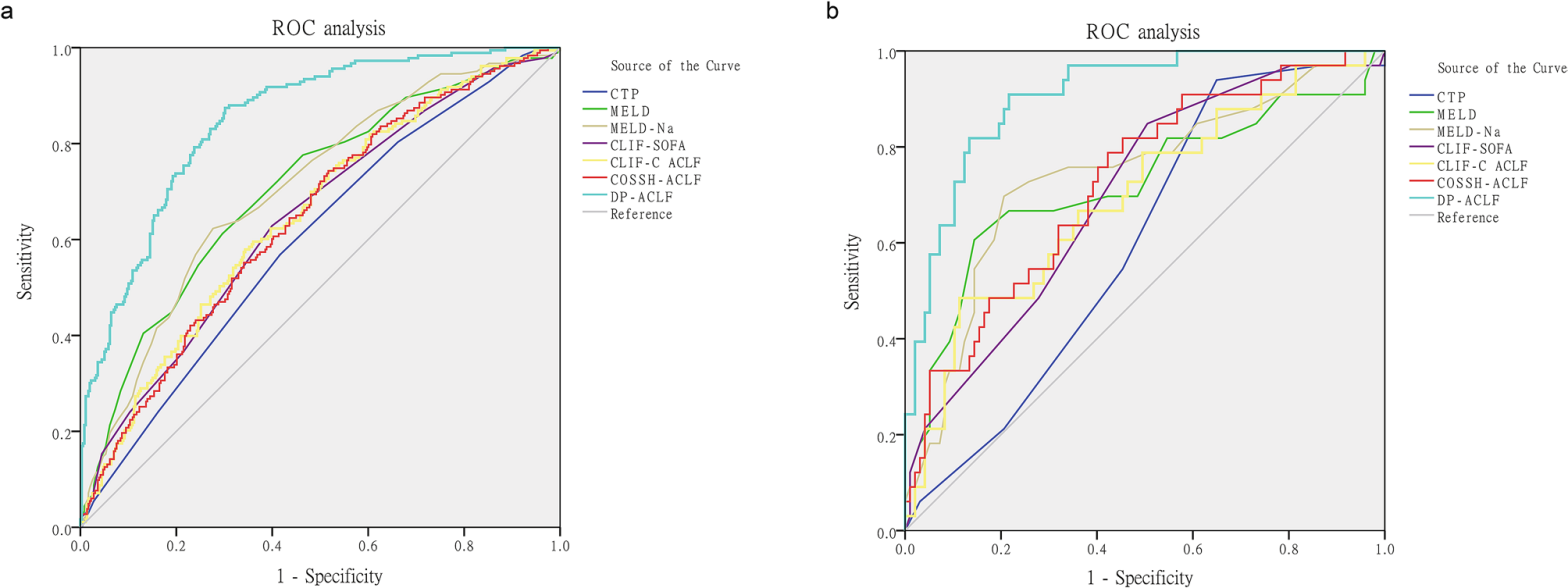
The DP-ACLF score was compared with the baseline data of other prognostic scoring systems, and ROC curves were performed to determine prognostic accuracy. (**a**) Training group. (**b**) Validation group.

The ROC analysis of all patients in the training set showed that the AUC values and accuracy of DP-ACLF and the baseline data of other prognostic scores were as follows: DP-ACLF (0.850/76.3%), CTP (0.601/66.2%), MELD (0.706/69.7%), MELD-Na (0.706/68.2%), CLIF-SOFA (0.645/68.4%), CLIF-C ACLF (0.651/66.5%), and COSSH-ACLF (0.644/66.7%). The DP-ACLF had significantly higher AUCs than other models (*p* < 0.001, respectively), and its predictive accuracy was the best (Table 5 and Fig. 5a).

**Table 5:** Training group: ROC correlation analysis and comparison of DP-ACLF and other prognostic scores.

Variable	AUC (95% CI)	St	Cut-off value	Sensitivity	Specificity	Positive likelihood ratio	Negative likelihood ratio	Accuracy	*z statistic (*p* value)
CTP	0.601 (0.558–0.643)	0.025	12	0.568	0.584	0.058	0.029	66.2%	8.616 (*p* < 0.001)
MELD	0.706 (0.666–0.744)	0.024	25	0.612	0.704	0.326	0.078	69.7%	6.777 (*p* < 0.001)
MELD-Na	0.706 (0.666–0.744)	0.023	28	0.623	0.724	0.376	0.122	68.2%	6.363 (*p* < 0.001)
CLIF-SOFA	0.645 (0.603–0.685)	0.024	8	0.628	0.601	0.181	0.047	68.4%	8.174 (*p* < 0.001)
CLIF-C ACLF	0.651 (0.610–0.692)	0.025	43	0.579	0.656	0.212	0.091	66.5%	7.612 (*p* < 0.001)
COSSH-ACLF	0.644 (0.602–0.685)	0.025	6	0.743	0.483	0.151	0.062	66.7%	7.871 (*p* < 0.001)
DP-ACLF	0.850 (0.817–0.879)	0.017	9.4	0.874	0.698	1.259	0.151	76.3%	NA

### External validation of the DP-ACLF (validation set cases)

Overall, 142 cases of ACLF who met the inclusion and exclusion criteria were collected from Beijing You’an Hospital Affiliated to Capital Medical University. Three cases with incomplete data and nine cases who underwent liver transplantation within 90 days were further excluded. The remaining 130 patients constituted the validation group, in which 97 cases survived and 33 cases died within 90 days (Fig. 1). Supplementary Table S6 describes the baseline characteristics of these patients. Compared with the training group, the median age in the validation group was lower (*t* = 5.644, *p* < 0.001) and the proportion of male patients was higher (χ^2^ = 28.951, *p* = 0.003). The validation group also had a higher proportion of patients with a basic etiology due to HBV infection and alcoholic liver disease (χ^2^ = 210.991, *p* < 0.001). No significant differences were found between the training and validation groups with regard to 90-day mortality or requirement of ALSS. ROC analysis showed that the predictive accuracy of DP-ACLF on ACLF prognosis was better in the validation group, with an AUC value of 0.907 and an accuracy rate of 85.4%. These values were significantly higher than the baseline values of CTP (0.601/74.6%), MELD (0.721/76.2%), MELD-Na (0.740/73.8%), CLIF-SOFA (0.701/76.9%), CLIF-C ACLF (0.694/74.6%), and COSSH-ACLF (0.724/77.7%) (*p* < 0.001, respectively) (Table 6 and Fig. 5b).

**Table 6:** Validation group: ROC correlation analysis and comparison of DP-ACLF and other prognostic scores.

Variable	AUC (95% CI)	St	Cut-off value	Sensitivity	Specificity	Positive likelihood ratio	Negative likelihood ratio	Accuracy	*z statistic (*p* value)
CTP	0.601 (0.511–0.685)	0.051	11	0.939	0.351	0.031	0.010	74.6%	5.647 (*p* < 0.001)
MELD	0.721 (0.636–0.796)	0.059	26	0.606	0.856	0.269	0.054	76.2%	3.637 (*p* < 0.001)
MELD-Na	0.740 (0.655–0.813)	0.054	27	0.697	0.794	0.222	0.078	73.8%	3.571 (*p* < 0.001)
CLIF-SOFA	0.701 (0.615–0.778)	0.050	8	0.849	0.495	0.138	0.010	76.9%	4.596 (*p* < 0.001)
CLIF-C ACLF	0.694 (0.607–0.771)	0.055	48	0.485	0.887	0.138	0.043	74.6%	4.534 (*p* < 0.001)
COSSH-ACLF	0.724 (0.639–0.799)	0.051	6	0.788	0.577	0.320	0.043	77.7%	4.446 (*p* < 0.001)
DP-ACLF	0.907 (0.843–0.951)	0.027	9.7	0.909	0.784	1.75	0.078	85.4%	NA

**Figure 6:**
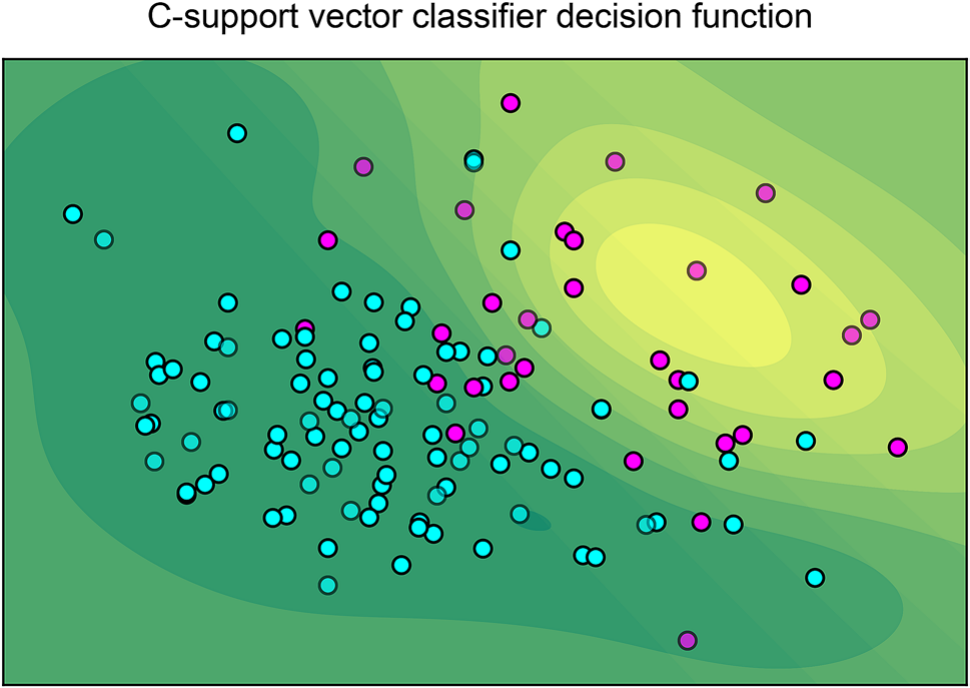
According to the DP-ACLF scores, C-support vector classifier function was used to predict the decision function value of each case in the validation group. Python software with matplotlib and sklearn packages was used to show that 90 cases of survivors (blue) (survival time ≥ 90 days) and 21 cases of non-survivors (red) (survival time < 90 days) were distributed in the green and yellow areas, respectively. The darker the color of the region in which the dots were located, the more accurate the prediction was.

Based on the DP-ACLF scores, C-support vector classifier function was used to predict the decision function value of each case. Python software with sklearn packages revealed that DP-ACLF had an excellent ability to discriminate survival and non-survival cases (Fig. 6). Univariate Cox analysis was used to evaluate the model fit of each prognostic model for the survival time and state of patients. The results showed that the Harrell's C and Somer's D values of each model were as follows: DP-ACLF (0.877/0.754), CTP (0.580/0.160), MELD (0.700/0.400), MELD-Na (0.715/0.430), CLIF-SOFA (0.683/0.366), CLIF-C ACLF (0.683/0.366), and COSSH-ACLF (0.714/0.428). Harrell's C and Somer's D indexes for DP-ACLF were higher than for other models (Table 7).

**Table 7:** Validation group: univariate Cox regression analysis of survival time and ACLF status with DP-ACLF and other prognostic scoring systems.

Variable	*p* value	Hazard ratio (95% CI)	Harrell’s C (95% CI)	Somer’s D (95% CI)
CTP	0.106	1.214 (0.959–1.540)	0.580 (0.504–0.664)	0.160 (0.008–0.328)
MELD	*p* < 0.001	1.148 (1.079–1.220)	0.700 (0.603–0.799)	0.400 (0.206–0.598)
MELD-Na	*p* < 0.001	1.072 (1.038–1.108)	0.715 (0.626–0.805)	0.430 (0.252–0.610)
CLIF-SOFA	*p* < 0.001	1.612 (1.270–2.047)	0.683 (0.630–0.773)	0.366 (0.260–0.546)
CLIF-C ACLF	*p* < 0.001	1.093 (1.042–1.147)	0.683 (0.590–0.780)	0.366 (0.180–0.560)
COSSH-ACLF	*p* < 0.001	2.736 (1.799–4.162)	0.714 (0.630–0.800)	0.428 (0.260–0.600)
DP-ACLF	*p* < 0.001	2.564 (2.008–3.274)	0.877 (0.832–0.923)	0.754 (0.664–0.846)

**Figure 7:**
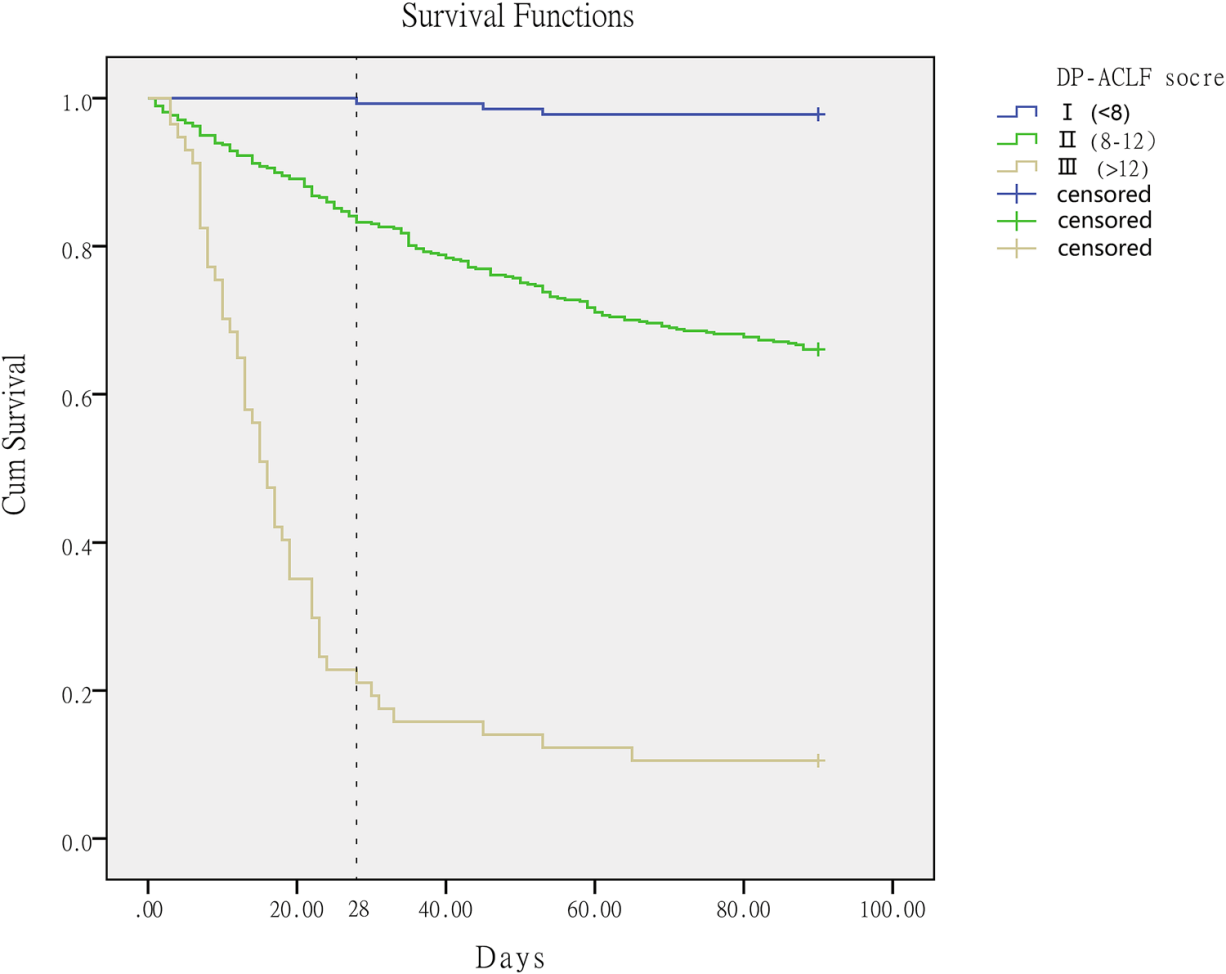
Kaplan–Meier survival analysis showing a significant difference in 28-day (χ^2^ = 238.411, *p* < 0.001) and 90-day cumulative survival rates (χ^2^ = 270.194, *p* < 0.001) in patients with ACLF who were graded according to the DP-ACLF score.

The DP-ACLF scores in the two groups ranged from 4 to 15. The 28-day and 90-day survival rates of patients with different DP-ACLF scores can be found in the supplementary Figure S2. When categorizing ACLF patients into grade I (DP-ACLF < 8), grade II (DP-ACLF between 8 and 12), and grade III (DP-ACLF > 12), Kaplan–Meier analysis showed that the 90-day cumulative survival rate was 97.8% for grade I patients, 66.0% for grade II patients, and 10.5% for grade III patients; these differences were all statistically significant (χ^2^ = 270.194, *p* < 0.001). Additionally, 28-day cumulative survival rates were also significantly different in grade I, II, and III patients (99.3%, 83.2%, and 21.1%, respectively) (χ^2^ = 238.411, *p* < 0.001) (Fig. 7).

## Discussion

### Main findings

Using a large multi-center group of ACLF patients (training set), we identified that age, WGO type, basic etiology, and clinical indicators such as TB, Cr, PTA, and HE stage were independent prognostic factors in ACLF. Furthermore, we found that the dynamic trends of TB, Cr, PTA, and HE stage were also independent prognostic factors. On this basis, we constructed a new logistic dynamic prognosis prediction model: DP-ACLF. This model showed better predictive efficacy than other prognostic scoring systems at different time points. Classifications of ACLF based on DP-ACLF helped discriminate patients concerning 28- and 90-day mortalities.

### Comparison with previous research

In our study, we confirmed that prognosis varied depending on WGO ACLF type, and type C was a risk factor for short-term mortality in ACLF. A recent Chinese research on the tri-typing of HBV-ACLF in accordance with the WGO definition also showed that type-C ACLF obtained the highest 28-day (65.2%) and 90-day (75.3%) mortalities, compared with type-A (48.7% and 54.4%, respectively) and type-B (48.4% and 62.8% respectively) ACLF cases; tri-typing of HBV-ACLF was able to distinguish clinical characteristics, including precipitating events, organ failure, and short-term prognosis in ACLF patients^20^.

In addition, stratified analysis based on WGO type revealed significant prognostic differences in type C ACLF with regard to etiology (HBV infection or alcohol). The MELD score^6,15^ included the basic etiology in the calculation formula, patients with cholestasis and alcoholic liver disease might have better prognoses than those with other etiologies. It was also suggested that liver failure due to viral hepatitis might have a poorer prognosis. However, in type A and B ACLF in our study, the prognosis of HBV-ACLF was not significantly worse than that of ACLF with alcoholic liver disease. The prognostic impact of different etiologies, such as HBV infection, alcohol, autoimmune, or other underlying liver diseases, were also not found in the APASL-AARC guidelines, in which the diagnostic criteria for ACLF only include non-cirrhotic or compensated cirrhosis patients^21^. Therefore, the effect of etiology on short-term prognosis might be more pronounced in type C ACLF.

Due to significant differences in the definition of ACLF between Eastern and Western countries, the underlying etiology, precipitating events, and prognosis of ACLF differ in different regions^22^. Therefore, many researches recently had been devoted to the development of diagnostic criteria and prognostic models for single-etiology ACLF. The COSSH study from China, for example, provided a useful exploration of the diagnostic criteria and prognosis of HBV-ACLF^17^. However, there is still a need in clinical practice for prognostic models that can identify high-risk ACLF populations in patients with different etiologies and states of chronic liver diseases. In this study, both WGO type and basic etiology were included in the new model, which significantly expanded the clinical applicability of the model.

ALSS is an important treatment for patients with ACLF, however, the results of current studies on whether treatment with ALSS can improve the prognosis of ACLF are not consistent. Andreas et al.^23^ conducted a prospective study in 10 university hospitals in 7 European countries and found that the prometheus system did not improve the short-term prognosis of ACLF patients, but it could improve the 90-day survival rate of ACLF patients with MELD > 30 points. A meta-analysis that included 25 randomized controlled studies of 738 patients with ALF and 1040 with ACLF found that ALSS reduced mortality by 16% in patients with liver failure, with a particularly significant effect on ACLF^24^. However, in this study, due to the fact that only 37.9% of cases were treated with ALSS, the sample size was small, and there were numerous factors influencing the prognosis of ACLF, resulting in failure to find a statistically significant difference in the effect of ALSS on prognosis. In the future, a definitive stratified analysis of ACLF patients with a single cause, same severity and stage may help to further clarify the role of ALSS interventions on the prognosis.

It is well known that ACLF is a dynamic syndrome which is reversible in a considerable proportion of patients, and this feature is closely related to improvement of prognosis, suggesting the importance of sequential dynamic assessment^25,26^. Moreau et al.^7^ reported that when the CLIF-C ACLF score was computed at 48 h, 3–7 days, and 8–15 days after the diagnosis of ACLF, the predictive accuracy of 28-day mortality was significantly better than when the score was calculated at the diagnosis. Many studies had chosen the 3rd to 7th day after diagnosis as the evaluation time-point. For example, previous research reported that there were significant differences in clinical characteristics and risk factors of ACLF between day 1 and 7, and ACLF classification at days 3–7, rather than at diagnosis, was the best predictor of prognosis^9^. However, it had also been argued that the duration of the ACLF should be 12 weeks, since the data on ACLF in patients with cirrhosis who had undergone surgery showed a 12-week period of increased risk of mortality^2^. Clinically, some ACLF patients either recover or deteriorate rapidly within 3–7 days, while others show a protracted course, with good or poor prognosis in 1–3 months; those patients with late deterioration still need to be considered for liver transplantation. Thus, much attention should be paid to changes occurring in ACLF at days 3–7 after admission, as well as the other time points; this would not only help determine prognosis, but also aid in the proper design of liver transplantation protocols. For instance, it had been reported that change in MELD score at the second week provided an early opportunity for prognostication in ACLF. A MELD score that did not deteriorate by week 2 would predict 93.8% chance of survival for the next 60 days^27^.

Although the levels of clinical indicators such as TB, Cr, PTA, and HE at diagnosis were closely correlated with ACLF prognosis, and were relatively common in previous ACLF scoring systems, their changing trends during clinical course were also important determinants of short-term mortality. Herein, the dynamic trends of ACLF indicators were combined with baseline data to obtain a new model, DP-ACLF, which could accurately reflect the clinical course and the responsiveness to medical treatment. At the third day, first week, and second week after admission for patients with ACLF, the short-term prognostic performance of DP-ACLF was superior to other prognostic scores, including CTP, MELD, MELD-Na, CLIF-SOFA, CLIF-C ACLF, and COSSH-ACLF. And for patients with survived time longer than 2 weeks, the second week of DP-ACLF score had the highest predictive efficacy. Additionally, classification of ACLF into grades I, II, and III based on the DP-ACLF score could be evaluated to discriminate patients concerning 28- and 90-day mortalities more accurately, and thus facilitated timely clinical decision-making and healthcare resource allocation.

### Limitations

This was a retrospective study and selective bias might be present. However, its multicentric nature, objective inclusion and exclusion criteria, and low data loss helped to mitigate the potential for such bias.

## Conclusion

In patients with ACLF, initial baseline characteristics as well as dynamic trends in clinical indicators are benefit for predicting clinical course and short-term prognosis. The dynamic prediction model proposed in this study was superior to other established scoring models in predicting ACLF prognosis. Prospective studies should be conducted to further validate the predictive performance of the DP-ACLF. We hope our findings may help clinicians to better identify high-risk ACLF patients and optimize clinical decision-making.

## Supplementary Information

Supplementary Information.

## Abbreviations

ACLF: Acute-on-chronic liver failure
CTP: Child–Turcotte–Pugh
MELD: Model for End-stage Liver Disease score
CLIF-SOFA: Chronic Liver Failure Sequential Organ Failure Assessment score
CLIF-C ACLF: Chronic Liver Failure Consortium Acute-on-Chronic Liver Failure score
APASL: Asian Pacific Association for the Study of the Liver
AARC: APASL ACLF Research Consortium
WGO: World Gastroenterology Organization
TB: Serum total bilirubin
INR: International normalized ratio
PTA: Prothrombin activity
ALSS: Artificial liver support system
MELD-Na: MELD-sodium
HBV: Hepatitis B virus
HBV-ACLF: Hepatitis B virus-related ACLF
COSSH: Chinese Group on the Study of Severe Hepatitis B
AUC: Area under the ROC curve
Cr: Serum creatinine
HE: Hepatic encephalopathy
DP-ACLF: The dynamic prediction model for prognosis of ACLF
SDTs: Sum of dynamic trend score
