# Supplementary material for: A dynamic prediction model for prognosis of acute-on-chronic liver failure based on the trend of clinical indicators
Source: Sci Rep. 2021 Jan 19;11:1810. doi: 10.1038/s41598-021-81431-0 (PMC7815739; doi:10.1038/s41598-021-81431-0)
Supplement: Supplementary file 1 — Supplementary Information. [file 41598_2021_81431_MOESM1_ESM.docx]

**A dynamic prediction model for prognosis of acute-on-chronic liver failure based on the trend of clinical indicators**

Zhenjun Yu^#1^, Yu Zhang^#1^, Yingying Cao^1^, Manman Xu^2^, Shaoli You^3^, Yu Chen^2^, Bing Zhu^3^, Ming Kong^2^, Fangjiao Song^3^, Shaojie Xin*^3^, Zhongping Duan*^2^, Tao Han*^145^

**Supplementary Tables:**

Table S1. Training Set, Baseline clinical characteristics of patients on admission.

| Baseline Characteristics | Overall (n=541) |
| --- | --- |
| Age(years) | 50 (43-59) |
| Sex (male) | 414 (76.5%) |
| Compensated Cirrhosis | 219 (40.5%) |
| Decompensated Cirrhosis | 234 (43.3%) |
| Etiology |  |
| Hepatitis B virus infection | 303 (56.1%) |
| Alcoholism | 102 (18.9%) |
| Hepatitis B virus infection and Alcoholism | 45 (8.3%) |
| Others | 91 (16.8%) |
| Precipitant event |  |
| Hepatitis B virus reactivation | 39 (7.2%) |
| Alcoholism | 14 (2.6%) |
| Bacterial Infections | 96 (17.7%) |
| Drugs or Poisons | 46 (8.5%) |
| Others | 50 (9.2%) |
| Unknow | 296 (54.7%) |
| Complication |  |
| Hepatic encephalopathy | 112 (20.7%) |
| Gastrointestinal bleeding | 99 (18.3%) |
| Ascites | 418 (77.3%) |
| Abdominal infection | 218 (40.3%) |
| Pulmonary infection | 128 (23.7%) |
| Biliary infection | 20 (3.7%) |
| Intestinal infection | 21 (3.9%) |
| Laboratory |  |
| White blood cell (×10^9^/L) | 6.7 (4.7-9.1) |
| Neutrophil ratio (%) | 72.0 (63.1-79.8) |
| Hemoglobin (g/L) (n=535) | 118 (102-136) |
| Platelet (×10^9^/L) | 87 (56-126) |
| C-reactive protein (mg/L) (n=190) | 14.4 (9.2-25.0) |
| Alanine aminotransferase (U/L) (n=539) | 132.0 (43.0-461.0) |
| Aspartate aminotransferase (U/L) (n=532) | 180.5 (90.5-471.8) |
| Alkaline phosphatase (U/L) | 142.0 (111.5-185.5) |
| Glutamate transpeptidase (U/L) | 87.0 (53.0-151.0) |
| Cholinesterase (IU/L) | 2599.5 (1788.8-3507.5) |
| Albumin (g/L) | 28.7 (25.0-32.0) |
| Globulin (g/L) | 30.9 (25.7-36.5) |
| Total Bilirubin (mg/dL) | 13.9 (8.7-20.4) |
| Blood urea nitrogen (mmol/L) | 5.1 (3.6-9.0) |
| Creatinine (mg/dL) | 0.9 (0.7-1.1) |
| Serum sodium (mmol/L) | 135.0 (131.4-137.7) |
| Serum potassium (mmol/L) | 3.9 (3.5-4.3) |
| Serum chloride (mmol/L) (n=538) | 99.9 (95.7-103.1) |
| Triglyceride (mmol/L) (n=288) | 1.0 (0.7-1.4) |
| Total cholesterol (mmol/L) (n=291) | 1.7 (1.1-2.4) |
| Prothrombin Activity (%) | 36 (29-45) |
| Alpha-fetoprotein (ng/mL) (n=296) | 32.5 (5.5-121.0) |
| Serum ammonia (mmol/L) (n=418) | 61 (42-84) |
| Organ failures^#^ |  |
| Liver | 343 (63.4%) |
| Kidneys | 25 (4.6%) |
| Brain | 27 (5.0%) |
| Coagulation | 138 (25.5%) |
| Lungs | 20 (3.7%) |
| Circulation | 3 (0.6%) |
| Organ support |  |
| Artificial liver support system | 205 (37.9%) |
| Prognostic score |  |
| CTP | 11 (10-12) |
| MELD | 22 (18-26) |
| MELD-Na | 25 (19-31) |
| CLIF-SOFA | 7 (7-9) |
| CLIF-C ACLF | 40 (35-46) |
| COSSH-ACLF | 5.3 (4.8-5.8) |

^#^Organ failures were judged by reference to the CLIF-SOFA definition of organ failure[^1^](#_ENREF_1).

Table S2. Stratified analysis of the impact of artificial liver support system (ALSS) treatment on the short-term prognosis of ACLF in the training set.

| Variable | | With ALSS treatment (n) (Non-survivor (n)), % | Without ALSS treatment (n) (Non-survivor (n)), % | χ^2^ value | *p* value |
| --- | --- | --- | --- | --- | --- |
| Age (years) | ≥60 | 47 (18), 38.3% | 80 (34), 42.5% | 0.216 | 0.642 |
|  | <60 | 158 (56), 35.4% | 256 (75), 29.3% | 1.706 | 0.191 |
| Sex | male | 159 (59), 37.1% | 255 (78), 30.6% | 1.880 | 0.170 |
|  | female | 46 (15), 32.6% | 81 (31), 38.3% | 0.407 | 0.523 |
| WGO type | Type A | 45 (13), 28.9% | 43 (9), 20.9% | 0.743 | 0.389 |
|  | Type B | 89 (25), 28.1% | 130 (32), 24.6% | 0.331 | 0.565 |
|  | Type C | 71 (36), 50.7% | 163 (68), 41.7% | 1.618 | 0.203 |
| Etiology | Alcoholic | 38 (10), 26.3% | 64 (13), 20.3% | 0.492 | 0.483 |
|  | HBV | 116 (46), 39.7% | 186 (66), 35.5% | 0.533 | 0.465 |
|  | HBV and Alcoholic | 19 (10), 52.6% | 26 (10), 38.5% | 0.893 | 0.345 |
| Combined infection | Yes | 144 (56), 38.9% | 196 (70), 35.7% | 0.359 | 0.549 |
|  | No | 61 (18), 29.5% | 140 (39), 27.9% | 0.057 | 0.811 |
| MELD  score | >30 | 22 (12), 54.5% | 39 (26), 66.7% | 0.880 | 0.348 |
|  | ≤30 | 183 (62), 33.9% | 297 (83), 27.9% | 1.891 | 0.169 |

Table S3. The third-day variation range of Total Bilirubin, Creatinine, Prothrombin Activity and Hepatic Encephalopathy was set at 10-50%, with Univariate Cox regression analysis to determine the survival model fit.

| The 3^rd^ day | Group setting | *p* value | Hazard ratio (95%CI) | Harrell’s C (95%CI) |
| --- | --- | --- | --- | --- |
| Total Bilirubin trend | Boundary setting (10%) | *p* <0.001 | 1.735(1.32-2.282) | 0.560(0.532-0.588) |
|  | Boundary setting (20%) | *p* <0.001 | 1.725(1.389-2.144) | 0.586(0.554-0.618) |
|  | Boundary setting (30%) | *p* <0.001 | 1.673(1.395-2.007) | 0.606(0.571-0.641) |
|  | Boundary setting (40%) | *p* <0.001 | 1.824(1.303-2.553) | 0.543(0.519-0.567) |
|  | Boundary setting (50%) | *p* =0.002 | 1.882(1.264-2.804) | 0.532(0.511-0.554) |
| Creatinine trend | Boundary setting (10%) | *p* =0.002 | 1.386(1.132-1.697) | 0.562(0.527-0.599) |
|  | Boundary setting (20%) | *p* <0.001 | 1.622(1.259-2.088) | 0.564(0.531-0.598) |
|  | Boundary setting (30%) | *p* <0.001 | 1.649(1.218-2.231) | 0.557(0.528-0.586) |
|  | Boundary setting (40%) | *p* =0.010 | 1.626(1.121-2.359) | 0.531(0.505-0.555) |
|  | Boundary setting (50%) | *p* =0.002 | 1.933(1.288-2.901) | 0.533(0.510-0.556) |
| Prothrombin Activity trend | Boundary setting (10%) | *p* =0.003 | 1.677(1.195-2.354) | 0.542(0.514-0.570) |
|  | Boundary setting (20%) | *p* <0.001 | 1.596(1.247-2.043) | 0.565(0.532-0.597) |
|  | Boundary setting (30%) | *p* <0.001 | 1.387(1.157-1.662) | 0.567(0.531-0.604) |
|  | Boundary setting (40%) | *p* =0.09 | 1.472(0.939-2.309) | 0.520(0.499-0.541) |
|  | Boundary setting (50%) | *p* =0.25 | 1.366(0.801-2.331) | 0.512(0.496-0.529) |
| Hepatic Encephalopathy trend (West Haven stage) | Boundary setting (1 level) | *p* =0.002 | 2.345 (1.363-4.034) | 0.521(0.503-0.538) |
|  | Boundary setting (2 level) | *p* =0.611 | 1.293 (0.481-3.478) | 0.501(0.493-0.510) |
|  | Boundary setting (3 level) | *p* =0.097 | 5.307 (0.741-38.001) | 0.502(0.498-0.506) |

Table S4. The first-week variation range of Total Bilirubin, Creatinine, Prothrombin Activity and Hepatic Encephalopathy was set at 10-50%, with Univariate Cox regression analysis to determine the survival model fit.

| The 1^st^ week | Group setting | *p* value | Hazard ratio (95%CI) | Harrell’s C (95%CI) |
| --- | --- | --- | --- | --- |
| Total Bilirubin trend | Boundary setting (10%) | *p* <0.001 | 1.905(1.596-2.274) | 0.650(0.613-0.686) |
|  | Boundary setting (20%) | *p* <0.001 | 2.096(1.713-2.564) | 0.651(0.615-0.686) |
|  | Boundary setting (30%) | *p* <0.001 | 2.2(1.749-2.767) | 0.649(0.609-0.685) |
|  | Boundary setting (40%) | *p* <0.001 | 2.214(1.712-2.862) | 0.627(0.598-0.658) |
|  | Boundary setting (50%) | *p* <0.001 | 1.988(1.462-2.703) | 0.585(0.557-0.614) |
| Creatinine trend | Boundary setting (10%) | *p* =0.003 | 1.327(1.101-1.6) | 0.569(0.529-0.609) |
|  | Boundary setting (20%) | *p* <0.001 | 1.752(1.378-2.227) | 0.597(0.558-0.636) |
|  | Boundary setting (30%) | *p* <0.001 | 1.942(1.461-2.582) | 0.593(0.556-0.629) |
|  | Boundary setting (40%) | *p* <0.001 | 1.975(1.433-2.723) | 0.565(0.530-0.599) |
|  | Boundary setting (50%) | *p* <0.001 | 2.54(1.811-3.563) | 0.571(0.540-0.603) |
| Prothrombin Activity trend | Boundary setting (10%) | *p* <0.001 | 1.965(1.487-2.597) | 0.590(0.553-0.625) |
|  | Boundary setting (20%) | *p* <0.001 | 1.668(1.334-2.086) | 0.595(0.556-0.634) |
|  | Boundary setting (30%) | *p* <0.001 | 1.582(1.319-1.897) | 0.597(0.556-0.638) |
|  | Boundary setting (40%) | *p* =0.002 | 1.837(1.272-2.652) | 0.558(0.528-0.589) |
|  | Boundary setting (50%) | *p* =0.102 | 1.412(0.934-2.135) | 0.523(0.509-0.547) |
| Hepatic Encephalopathy trend (West Haven stage) | Boundary setting (1 level) | *p* =0.044 | 1.695(0.999-2.874) | 0.519(0.498-0.541) |
|  | Boundary setting (2 level) | *p* =0.600 | 2.066(0.971-4.398) | 0.511(0.496-0.525) |
|  | Boundary setting (3 level) | *p* =0.052 | 3.991(0.99-16.093) | 0.505(0.497-0.512) |

Table S5. The second-week variation range of Total Bilirubin, Creatinine, Prothrombin Activity and Hepatic Encephalopathy was set at 10-50%, with Univariate Cox regression analysis to determine the survival model fit.

| The 2^nd^ week | Group setting | *p* value | Hazard ratio (95%CI) | Harrell’s C (95%CI) |
| --- | --- | --- | --- | --- |
| Total Bilirubin trend | Boundary setting (10%) | *p* <0.001 | 2.004(1.64-2.449) | 0.643(0.611-0.677) |
|  | Boundary setting (20%) | *p* <0.001 | 1.867(1.58-2.206) | 0.654(0.620-0.688) |
|  | Boundary setting (30%) | *p* <0.001 | 2.01(1.671-2.418) | 0.656(0.623-0.691) |
|  | Boundary setting (40%) | *p* <0.001 | 2.216(1.767-2.78) | 0.636(0.605-0.667) |
|  | Boundary setting (10%) | *p* <0.001 | 2.004(1.64-2.449) | 0.643(0.611-0.677) |
| Creatinine trend | Boundary setting (10%) | *p* <0.001 | 2.033(1.545-2.676) | 0.592(0.556-0.628) |
|  | Boundary setting (20%) | *p* <0.001 | 1.746(1.384-2.202) | 0.599(0.561-0.634) |
|  | Boundary setting (30%) | *p* <0.001 | 1.628(1.334-1.987) | 0.604(0.566-0.642) |
|  | Boundary setting (40%) | *p* <0.001 | 2.162(1.598-2.925) | 0.581(0.546-0.615) |
|  | Boundary setting (50%) | *p* <0.001 | 2.514(1.816-3.48) | 0.580(0.546-0.613) |
| Prothrombin Activity trend | Boundary setting (10%) | *p* <0.001 | 1.871(1.537-2.277) | 0.625(0.587-0.662) |
|  | Boundary setting (20%) | *p* <0.001 | 2.292(1.802-2.914) | 0.625(0.589-0.660) |
|  | Boundary setting (30%) | *p* <0.001 | 1.772(1.495-2.1) | 0.630(0.592-0.666) |
|  | Boundary setting (40%) | *p* <0.001 | 2.193(1.634-2.944) | 0.587(0.555-0.620) |
|  | Boundary setting (50%) | *p* <0.001 | 2.024(1.422-2.879) | 0.560(0.533-0.589) |
| Hepatic Encephalopathy trend (West Haven stage) | Boundary setting (1 level) | *p* <0.001 | 2.078 (1.503-2.878) | 0.564(0.533-0.595) |
|  | Boundary setting (2 level) | *p* <0.001 | 2.445 (1.617-3.698) | 0.542(0.518-0.565) |
|  | Boundary setting (3 level) | *p* <0.001 | 4.703 (2.773-7.976) | 0.533(0.515-0.551) |

Table S6. Validation Set, Baseline clinical characteristics of patients on admission.

| Baseline Characteristics | Overall (n=130) |
| --- | --- |
| Age(years) | 44 (37-52) |
| Sex (male) | 115 (88.5%) |
| Compensated Cirrhosis | 82 (63.1%) |
| Decompensated Cirrhosis | 18 (13.8%) |
| Etiology |  |
| Hepatitis B virus infection | 99 (76.2%) |
| Alcoholism | 20 (15.4%) |
| Hepatitis B virus infection and Alcoholism | 4 (3.1%) |
| Others | 7 (5.4%) |
| Precipitant event |  |
| Hepatitis B virus reactivation | 18 (13.8%) |
| Alcoholism | 12 (9.2%) |
| Bacterial Infections | 9 (6.9%) |
| Drugs or poisons | 12 (9.2%) |
| Others | 3 (2.3%) |
| Unknow | 76 (58.5%) |
| Complication |  |
| Hepatic encephalopathy | 51 (39.2%) |
| Gastrointestinal bleeding | 8 (6.2%) |
| Ascites | 93 (71.5%) |
| Abdominal infection | 114 (87.7%) |
| Pulmonary infection | 41 (31.5%) |
| Biliary infection | 0 (0%) |
| Intestinal infection | 0 (0%) |
| Laboratory |  |
| White blood cell (×10^9^/L) | 6.8 (4.6-10.1) |
| Neutrophil ratio (%) | 70.0 (60.8-79.6) |
| Hemoglobin (g/L) | 122 (103-136) |
| Platelet (×10^9^/L) | 102.5 (69.8-144.3) |
| C-reactive protein (mg/L) (n=89) | 12.0 (6.0-23.0) |
| Alanine aminotransferase (U/L) | 150.1 (65.1-369.9) |
| Aspartate aminotransferase (U/L) | 150.0 (94.2-305.0) |
| Alkaline phosphatase (U/L) (n=120) | 133.1 (102.2-168.3) |
| Glutamate transpeptidase (U/L) | 85.9 (51.3-146.7) |
| Cholinesterase (IU/L) (n=119) | 2950 (1903-4164) |
| Albumin (g/L) | 30.4 (26.8-33.8) |
| Globulin (g/L) | 32.3 (27.3-38.5) |
| Total Bilirubin (mg/dL) | 20.9 (12.9-27.3) |
| Blood urea nitrogen (mmol/L) | 4.6 (3.4-6.3) |
| Creatinine (mg/dL) | 0.7 (0.5-0.9) |
| Serum sodium (mmol/L) | 135.6 (131.9-138.2) |
| Serum potassium (mmol/L) | 3.9 (3.5-4.2) |
| Serum chloride (mmol/L) (n=93) | 101.0 (96.4-103.1) |
| Triglyceride (mmol/L) (n=61) | 1.2 (0.8-1.5) |
| Total cholesterol (mmol/L) (n=91) | 2.6 (2.3-3.2) |
| Prothrombin Activity (%) | 33 (26-41) |
| Alpha-fetoprotein (ng/mL) (n=125) | 51.0 (7.2-169.1) |
| Serum ammonia (mmol/L) (n=117) | 80 (51-112) |
| Organ failures^#^ |  |
| Liver | 103 (79.2%) |
| Kidneys | 2 (1.5%) |
| Brain | 2 (1.5%) |
| Coagulation | 44 (33.8%) |
| Lungs | 2 (1.5%) |
| Circulation | 1 (0.8%) |
| Organ support |  |
| Artificial liver support system | 50 (38.5%) |
| Prognostic score |  |
| CTP | 11 (10-12) |
| MELD | 23 (20-28) |
| MELD-Na | 25 (20-35) |
| CLIF-SOFA | 8 (7-9) |
| CLIF-C ACLF | 42 (36-46) |
| COSSH-ACLF | 5.3 (4.8-6.1) |

^#^Organ failures were judged by reference to the CLIF-SOFA definition of organ failure[^1^](#_ENREF_1).

**
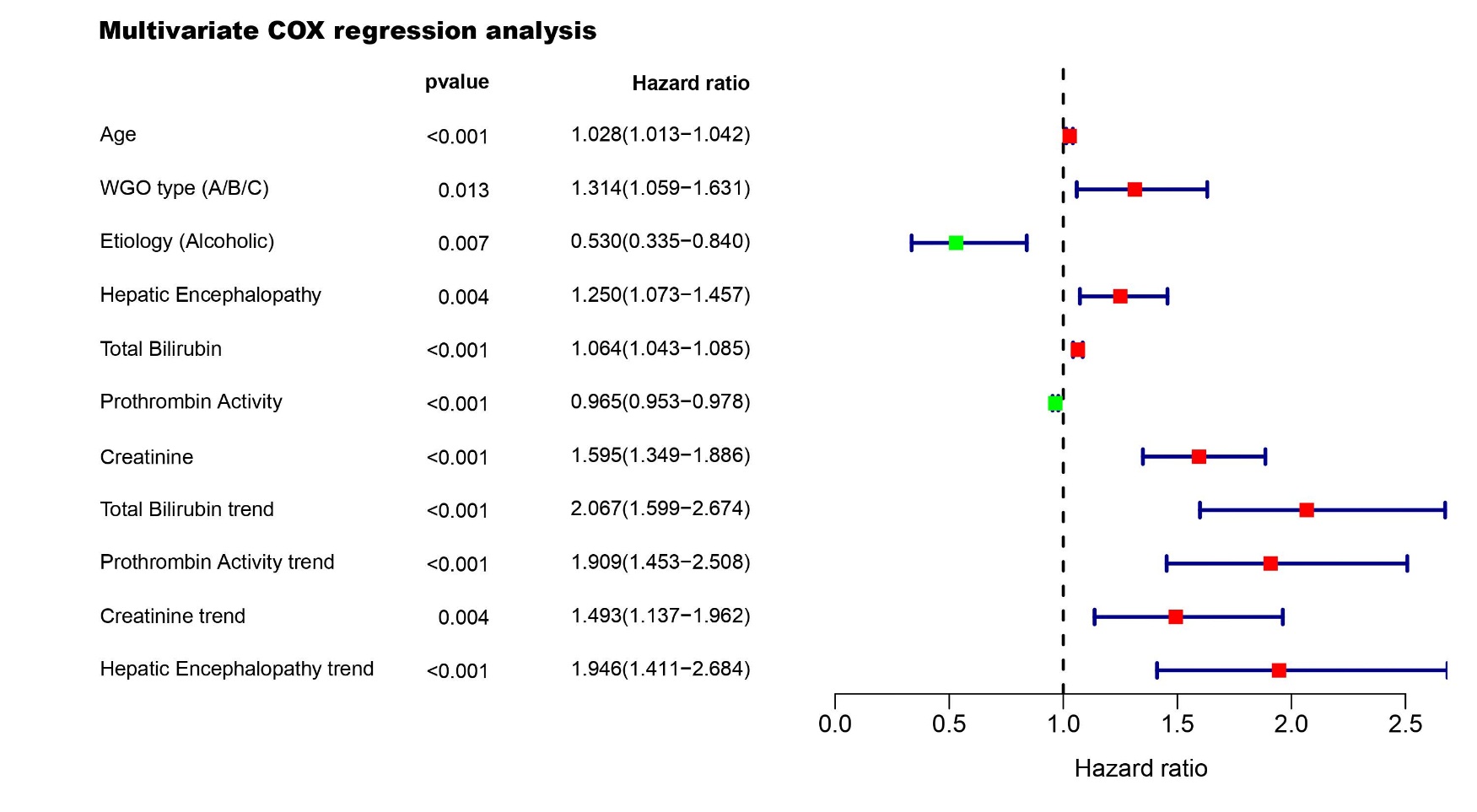
Supplementary Figures:**

Figure S1. Multivariate Cox Regression analysis showed that the dynamic trend scores of Total Bilirubin, Creatinine, Prothrombin Activity and Hepatic Encephalopathy were all independent prognostic factors of ACLF.


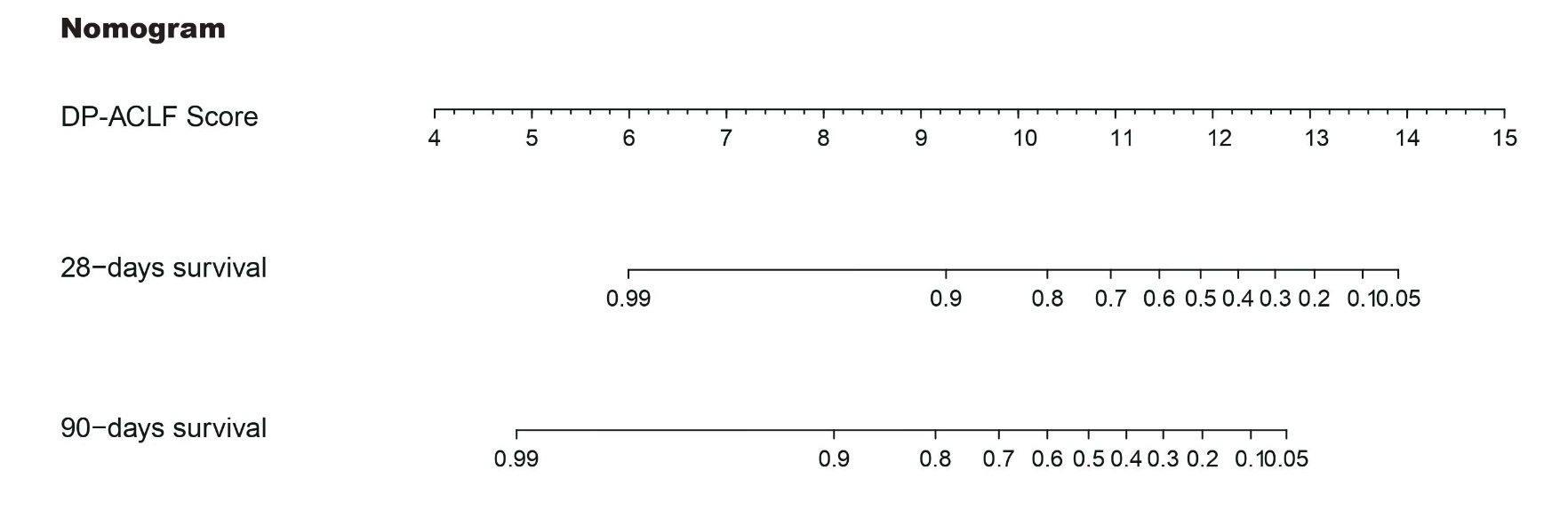


Figure S2. Nomogram showed the 28- and 90-day survival rates of ACLF with different DP-ACLF scores.

**Supplementary materials 1:**

Details of the prognostic scoring systems for patients with ACLF in this study.

1. Child-Turcotte-Pugh (CTP)[^2^](#_ENREF_2)

| CTP score | 1 | 2 | 3 |
| --- | --- | --- | --- |
| HE grade (West-Haven) | 0 | I~II | III~IV |
| Ascites | no | Mild | Moderate or Severe |
| TB(umol/L) | <34 | 34~51 | >51 |
| PT prolonged(s) | <4 | 4~6 | >6 |
| ALB(g/L) | >35 | 28~35 | <28 |

2. Chronic Liver Failure Sequential Organ Failure Assessment score (CLIF-SOFA) [^1^](#_ENREF_1)

| Organ/System | 0 | 1 | 2 | 3 | 4 |
| --- | --- | --- | --- | --- | --- |
| Liver (TB mg/dL) | <1.2 | 1.2~2.0 | 2.0~6.0 | 6.0~12.0 | >=12.0 |
| Kidney (Cr mg/dL) | <1.2 | 1.2~2.0 | 2.0~3.5 | 3.5~5.0 or Renal replacement therapy | >=5.0 |
| Nervous system (HE grade) | 0 | I | II | III | IV |
| Coagulation system (INR) | <1.1 | 1.1~1.25 | 1.25~1.5 | 1.5~2.5 | >=2.5 or PLT <=20×10^9^/L |
| Circulatory system (MAP mmHg or Antihypertensive drugs | >=70 | <70 | Dopamine <=5 or dobutamine or triopressin | Dopamine >5 or epinephrine<=0.1 or norepinephrine<=0.1 | Dopamine >15 or epinephrine>0.1 or norepinephrine>0.1 |
| Respiratory system (PaO_2_/FiO_2_ or SPO_2_/FiO_2_) | >400  >512 | 300~400  375~512 | 200~300  214~357 | 100~200  89~214 | <=100  <=89 |

3. Chronic Liver Failure Consortium Organ Function (CLIF-OF) [^3^](#_ENREF_3)

| Organ/System | 1 | 2 | 3 |
| --- | --- | --- | --- |
| Liver (TB mg/dL) | <6 | 6~12 | >12 |
| Kidney (Cr mg/dL) | <2 | 2~3.5 | >=3.5 or Renal replacement therapy |
| Nervous system (HE grade) | 0 | I~II | III~IV |
| Coagulation system (INR) | <2 | 2~2.5 | >=2.5 |
| Circulatory system (MAP mmHg) | >=70 | <70 | Antihypertensive drugs |
| Respiratory system (PaO_2_/FiO_2_  or SPO_2_/FiO_2_) | >300  >357 | 201~300  215~357 | <=200  <=214 |

4. Hepatitis B virus related Sequential Organ Failure Assessment score (HBV-SOFA)[^4^](#_ENREF_4)

| Organ/System | 1 | 2 | 3 |
| --- | --- | --- | --- |
| Kidney (Cr umol/L) | <103 | 104~206 | >206 |
| Nervous system (HE grade) | 0 | I~II | III~IV |
| Circulatory system (MAP mmHg) | >=70 | <70 | Antihypertensive drugs |
| Respiratory system (PaO_2_/FiO_2_  or SPO_2_/FiO_2_) | >300  >357 | 201~300  215~357 | <=200  <=214 |

5. The formula of MELD[^5^](#_ENREF_5) and MELD-sodium (MELD-Na)[^6^](#_ENREF_6) score: MELD = 3.78 × ln(TB (mg/dL)) + 11.2 × ln(INR) + 9.57 × ln(serum creatinine (mg/dL)) + 6.43× Etiology (the etiology was 0 for cholegenic or alcoholic, and 1 for others); MELD-Na = MELD+1.59 × (135-Na(mmol/L)).

6. The CLIF-SOFA score sums the severity grades of organ failures; and the CLIF-C ACLF score is a modification of the CLIF Consortium Organ Function (CLIF-OF) score: CLIF-C ACLF = 10 × (0.33 × CLIF-OF + 0.04 × age + 0.63 × ln(white blood cell(10^9^/L) – 2)[^1^](#_ENREF_1)^,^[^3^](#_ENREF_3).

7. The formula of COOSH-ACLF score: 0.741 × INR + 0.523 × HBV-SOFA + 0.026 × age + 0.003 × TB(mg/dL)[^4^](#_ENREF_4).

**References:**

1 Moreau, R. *et al.* Acute-on-chronic liver failure is a distinct syndrome that develops in patients with acute decompensation of cirrhosis. *Gastroenterology* **144**, 1426-1437. e1429 (2013).

2 Pugh, R., Murray‐Lyon, I., Dawson, J., Pietroni, M. & Williams, R. Transection of the oesophagus for bleeding oesophageal varices. *British journal of surgery* **60**, 646-649 (1973).

3 Jalan, R. *et al.* Development and validation of a prognostic score to predict mortality in patients with acute-on-chronic liver failure. *Journal of hepatology* **61**, 1038-1047 (2014).

4 Wu, T. *et al.* Development of diagnostic criteria and a prognostic score for hepatitis B virus-related acute-on-chronic liver failure. *Gut* **67**, 2181-2191 (2018).

5 Kamath, P. S. & Kim, W. R. The model for end‐stage liver disease (MELD). *Hepatology (Baltimore, Md.)* **45**, 797-805 (2007).

6 Biggins, S. W. *et al.* Evidence-based incorporation of serum sodium concentration into MELD. *Gastroenterology* **130**, 1652-1660, doi:10.1053/j.gastro.2006.02.010 (2006).
